# Does exhaled aerosol increase with COVID-19 infection correlate with body mass index-years?

**DOI:** 10.1073/pnas.2106088118

**Published:** 2021-06-29

**Authors:** Jürgen Stohner

**Affiliations:** ^a^Institute for Chemistry and Biotechnology (ICBT), Zurich University of Applied Sciences (ZHAW), CH-8820 Wädenswil, Switzerland;; ^b^ETH Zurich, Laboratory for Physical Chemistry, Hönggerberg, CH-8093 Zurich, Switzerland

**Fig. 1.**
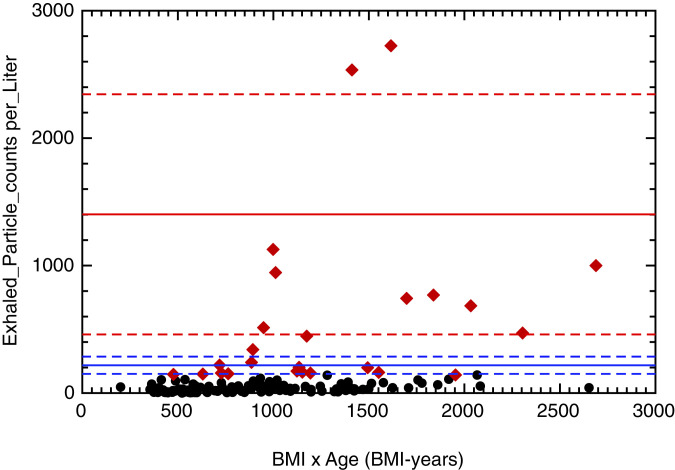
Exhaled particle counts per liter against BMI-years (black dots, low spreader; red dots, superspreader). Horizontal lines are the mean value (full line) for two superspreader cohorts (red and blue) and ±1 SD (dashed lines).

Edwards et al. (1) report on how exhaled aerosols influence COVID-19 infections. Respiratory droplet generation and exhalation were studied in human and nonhuman primate (NHP) subjects. The number of exhaled aerosol particles (NEAPs) varied by three orders of magnitude whereby the respiratory droplet number increases with COVID-19 infection and, presumably, with body mass index (BMI)-years. The authors divided the human cohort into three categories of superspreader (first and second decile) and low-spreader subjects. The NEAPs from every individual were graphically represented for each category (see figure 1 *B*–*D* in ref. 1) and show decreasing values along the x axis, thereby implying causality, whereas a randomized representation would be more adequate. To rationalize the categorization, we determined the mean values and the SDs [coverage factor k = 1, 68% confidence level (2)] for each of the three categories (1): Fig. 1*B* (mean value: 1,402; SD: 942), Fig. 1*C* (mean: 218; SD: 68), and Fig. 1*D* (mean: 42; SD: 37). The mean values for the two superspreader deciles (red and blue) are represented as horizontal lines and ±1 SD as dashed lines here in Fig. 1 (“Shewhart” plot). This analysis supports the authors’ categorization (1) with “typical” NEAPs which decrease roughly by an order of magnitude, but only at a low confidence level of 68% (±1 SD), whereas a coverage factor of 2 (95%) would already spoil this categorization.

**Fig. 2.**
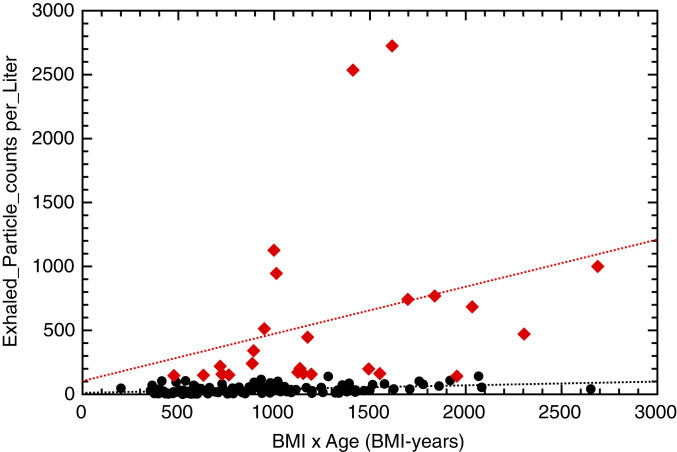
Exhaled particle counts per liter against BMI-years. The dotted lines are from a linear regression (see text).

The authors (1) conclude that “significant correlations were observed between exhaled aerosol, …, and particularly BMI-years.” Regression lines of the data are shown as red (two superspreader cohorts) and black (low spreader) dotted lines in Fig. 2. We are unable to reproduce the correlation coefficient ($r^{2}$ = 0.98) as given in the original caption to figure 2 in ref. 1.

The regression parameters obtained here (Fig. 2) are slope 11.6 (SD: 6.0; correlation coefficient: 0.40) for low spreaders (black) and slope 104.3 (SD: 347.1; correlation coefficient: 0.30) for superspreaders (red). The correlation coefficients are very small and do not support the authors’ view (1) about a significant correlation. For superspreaders (both deciles), the SD of the slope is much larger than the fitted value of the slope (already for k = 1). Therefore, we must draw the conclusion that there is no correlation between the number of exhaled particle counts per liter in humans and the BMI-years. The BMI-years of the NHPs are expected to be below 200, and a high number of exhaled particles per liter of 1,000 or more (see figure 4 in ref. 1) disfavors the claimed “strong correlation” even more.

Exploring the detailed role of aerosols in spreading severe acute respiratory syndrome coronavirus 2 is very important, but we do believe that this aspect of the complex issue of COVID-19 transmission and vulnerability of humans cannot be quantified with the aid of the BMI-years. The answer to the question raised in the title should therefore be no. There is no statistically relevant correlation in the data, but, even if it exists, this would not imply any causality.
